# Hydration monitoring and rehydration guidance system for athletes based on urine color’s L*a*b* parameters

**DOI:** 10.3389/fbioe.2022.1043028

**Published:** 2022-10-20

**Authors:** Yiwei Feng, Guoliang Fang, Minghai Li, Shuqiang Cui, Xue Geng, Chaoyi Qu, Jiexiu Zhao

**Affiliations:** ^1^ Exercise Biological Center, China Institute of Sport Science, Beijing, China; ^2^ School of Chemistry and Materials Science, Nanjing Normal University, Nanjing, China; ^3^ Beijing Institute of Sports Science, Beijing, China; ^4^ Department of Exercise Physiology, Beijing Sport University, Beijing, China

**Keywords:** athletes, hydration monitoring, rehydration guidance, urine color, color space, CIE L∗a∗b∗

## Abstract

Maintaining proper hydration is essential for athletes to sustain optimal performance and preserve their physical health. Existing studies have confirmed that urine color is one of the effective indicators for the subjective evaluation of athletes’ hydration through the urine color chart. However, the use of urine color charts to evaluate hydration is easily affected by the test environment, urine container and subjective feeling. At present, there are few hydration monitoring instruments based on quantitative analysis of urine color. In recent years, the L*a*b* color model has been widely used in the objective quantitative analysis of color. The L* value represents the luminance change from black to white, the a* value represents the chromaticity change from green to red, and the b* value represents the chromaticity change from blue to yellow. Our previous research has confirmed that the urine color b ∗ value is an effective new indicator to evaluate the hydration of athletes. The research team developed a urine hydration monitoring and rehydration guidance system based on the urine color’s L*a*b* parameters *via* wireless network technology and digital image technology. The hardware structure of the system is composed of a cuvette, a standard light source, a camera, an image collector, a host system, and a touch screen system. The system software is composed of functional modules, such as user information, image acquisition, image processing, and image recognition. The system operation process includes starting the system, filling in basic information, putting the sample, testing the sample, local data review, local data upload, and cloud data review. The system exhibits stable performance, a friendly operation interface, and simple and fast testing. It can objectively and accurately evaluate the hydration of athletes and provide personalized rehydration guidance. The system offers a new method for solving practical problems in sports training, and it has broad application prospects.

## 1 Introduction

Water is one of the essential nutrients for the human body. For the general public, dehydration may reduce thermoregulatory capacity, increase cardiovascular stress, and raise energy expenditure (McDermott et al., 2017). Long-term chronic hypohydration may increase the risk of kidney stones, urinary tract infections, constipation, adenomatous polyps, diabetes, and cardiovascular diseases (Manz, 2007). For athletes, maintaining proper hydration is essential to sustain optimal athletic performance, physiology, and health (Volterman and Moore, 2014). Dehydration of more than 1% of body weight can impair cognitive function; dehydration of more than 2% of body weight can reduce aerobic exercise capacity (McDermott et al., 2017). Dehydration greater than 3% of body weight can lead to exertional heat illnesses, such as heat cramps, heat exhaustion, and heat stroke (Casa et al., 2000). Acute overdrinking of water after exercise may lead to hyperhydration and increase the risk of exercise-induced hyponatremia (Hew-Butler et al., 2015).

Urine testing is a common noninvasive monitoring method that is highly accepted by athletes. Urine color, urine specific gravity (Usg), and urine osmolality are effective indicators for assessing hydration in athletes (Barley et al., 2020). In 1994, Armstrong et al. first developed a urine color chart and confirmed that it was an effective tool for the subjective assessment of urine color (Armstrong et al., 1994). However, the subjective assessment of urine color by using this chart is easily affected by many confounding factors, such as the test environment, test personnel, and urine container.

Another effective method is the quantitative testing of urine color through instruments. This method can prevent the results from being affected by the external environment and the operator’s subjectivity. The color space model is a mathematical model that quantitatively represents a certain color by using 1D, 2D, 3D, or even 4D model, such as the RGB, L*a*b*, and CMYK models. The L*a*b* model is a 3D color space model defined by the Commission Internationale de l´Eclairage (CIE) in 1976. This color model has the widest color gamut that contains all the colors visible to the human eyes. L* represents the change in brightness from black (0) to white (100), a* represents the color change from green (−128) to red (+127), and b* represents the change from blue (−128) to yellow (+127) (O'Brien et al., 1990). Recent studies have found that urine color can be objectively and quantitatively assessed by analyzing its L*a*b* parameters (Belasco et al., 2020). In addition, our research team previously confirmed that the b* value of urine color is an effective indicator for assessing the hydration of athletes by collecting 803 urine samples from athletes and testing urine color’s L*a*b* parameters, urine osmolality, and Usg. We found a strong correlation between urine color’s b* value and Usg (r = 0.811, *p* < 0.01), and a linear regression equation exists between them (b = −1,220 + 1,220 Usg, *R* ^2^ = 0.657) (Feng et al., 2022). Therefore, we envisioned that an instrument can be designed to monitor hydration by testing and quantifying urine color’s L*a*b* parameters.

In recent years, several research teams have developed hydration monitoring equipment based on the RGB space model of urine color through digital image technology. Digital image technology is a method that involves taking pictures with digital cameras or smartphones and then quantitatively analyzing the color information of the images to obtain the color parameters (Komatsu et al., 2016). Chin et al. designed an Internet of Things (IoT)-based pervasive body hydration tracker. This system measures the color of urine through its components, such as color sensors and urine test probes, and then uploads the results on a mobile phone. The phone infers hydration by analyzing the B value of the RGB space model of urine color (Chin and Tisan, 2015). Chew et al. (2020) evaluated hydration by taking pictures of urine with mobile phones and then verifying that the RGB values of the urine color captured by the mobile phones were highly correlated with the hydration status of dengue patients. However, urine color pictures taken with mobile phones are easily affected by the light source environment, shooting equipment, operators, and analysis software. Therefore, we envisage that developing a hydration monitoring device through digital image technology is feasible.

Currently, there are many emerging hydration monitoring devices such as patch antennas for monitoring hydration of the skin, sensors for measuring sodium ion (Na^+^) concentrations in sweat, micro-osmometers for measuring plasma osmolarity in 15 µl blood samples, and ultrasound systems for assessing hydration. These hydration monitoring devices are based on different testing indicators, and their designs and principles vary (Garrett et al., 2018). However, there are few studies on the urine hydration monitoring devices based on quantitative urine color analysis. Gunawan et al. (2018) developed a urine hydration system based on urine color and support vector machine. The system uses color sensor TCS34725 to obtain urine color data. The urine test results need to be accessed by Android smartphone, and the design of the detection device seems not rigorous enough. Chin et al. proposed an IoT-based pervasive body hydration tracker that consists of a urine color measurement component to be fitted in urinals. The test results need to be viewed through the Android smartphone, and the design of the device is complex (Chin and Tisan, 2015). Chew et al. (2020) captured urine color through the camera of the mobile phone, and then used Adobe Photoshop to process the picture. The study did not set test standards for mobile phone model, shooting environment and urine cup specification. The above monitoring methods are all based on RGB color space model to analyze urine color, and the practicability and accuracy of the equipment need more investigation. At present, no hydration monitoring system based on urine color’s L*a*b* parameters is available.

Based on the above background, our research team developed a urine hydration monitoring and rehydration guidance system to enrich the noninvasive monitoring method of athletes’ hydration and to serve during sports practice. This system combines sports nutrition, bioengineering, information technology, colorimetry, photometry, and other multidisciplinary theories. It uses urine color’s parameter b* value as the evaluation index for hydration, formulates a system hydration grading standard, adopts digital image technology, uses a camera to take urine images, and is combined with digital equipment to analyze the color space L*a*b* parameters.

This paper is organized as follows: In Section 2, we introduce the appearance, hardware and software design, design principle and operation process of the urine hydration monitoring system. Then, we report the experiment result and discuss the advantages, limitations and recommendations of the system in Section 3. Finally, some conclusions and prospects are drawn in Section 4.

## 2 Materials and methods

### 2.1 System design

**FIGURE 1:**
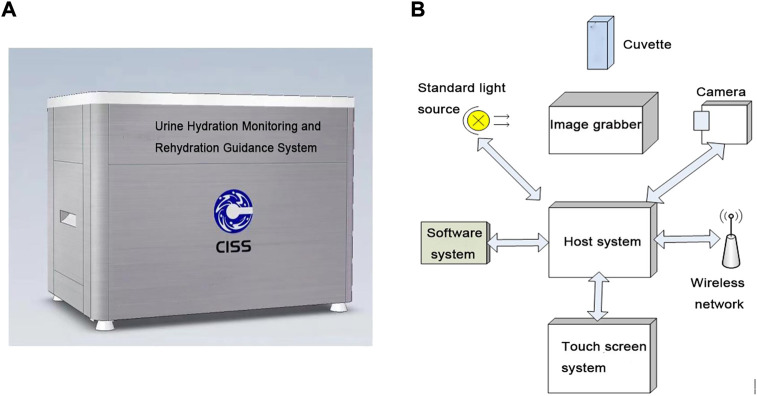
System design. **(A)** Schematic of the appearance of the system. **(B)** Schematic of system components.

The design of the system is presented in Figure 1A. The hardware part of the system is composed of a cuvette, a standard light source, a camera, a host system, a touch screen system, and an image collector. The software part is composed of a software system and a wireless network (Figure 1B).

#### 2.1.1 System hardware design

**FIGURE 2:**
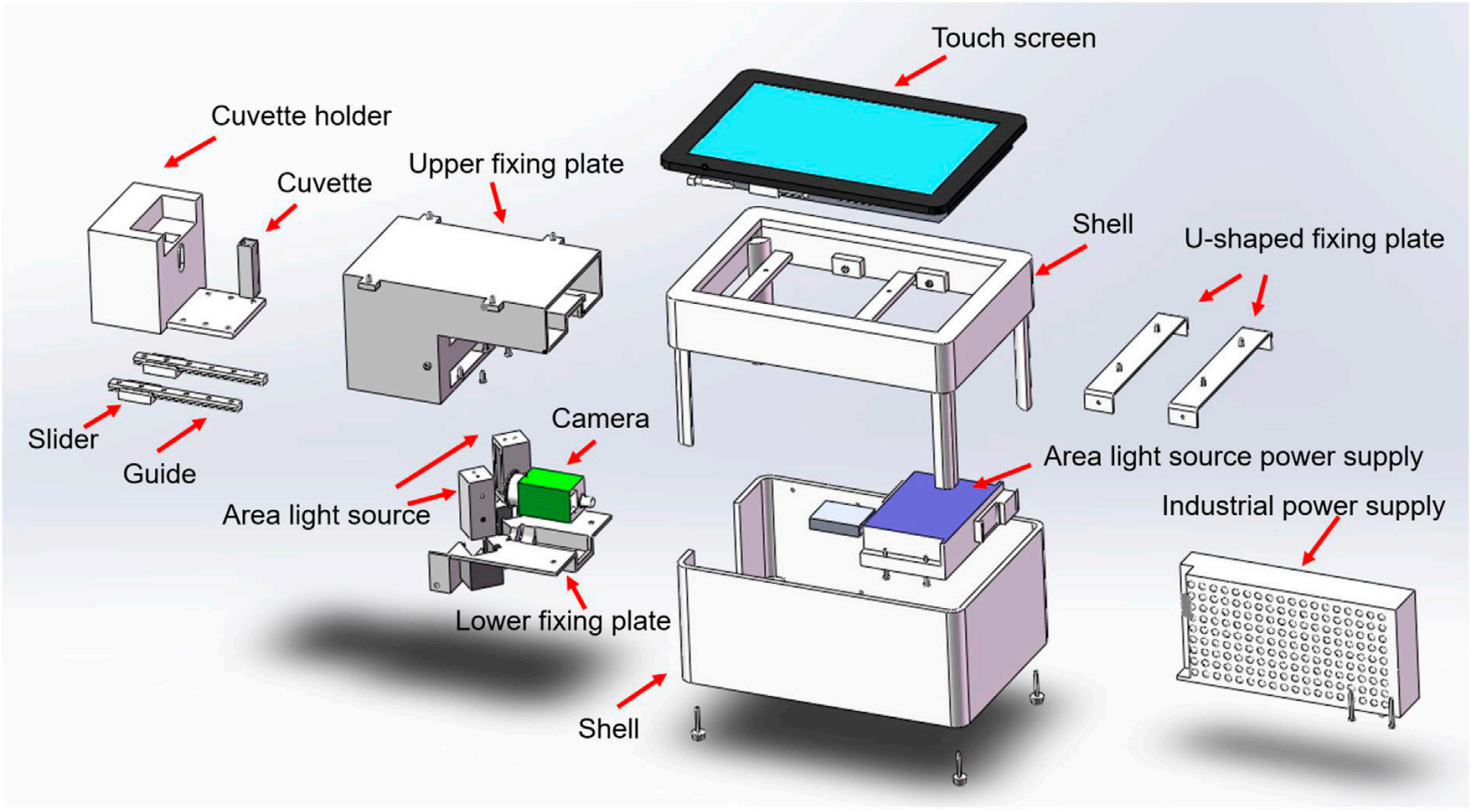
Schematic of the system hardware structure.

The hardware structure of this system is composed of a cuvette, a standard light source, a camera, an image collector, a host system, and a touch screen system (Figure 2). The structure and functions of the system’s hardware are as follows. 1) Cuvette: It is used to hold urine samples and is equipped with a sealing cover. We use a two-pass light quartz cuvette, which exhibits the characteristics of high light transmission and high acid and alkali resistance. 2) Standard light source: We use a multi-chip white light-emitting diode (LED) surface light source that can provide a uniform lighting environment for all the samples under different test environments. 3) Camera: We use a high-definition camera (model IR500RC-14) with more than 5 million pixels and IP67 waterproof design. This camera can be used for a long time. The captured image is displayed in true color, with a maximum of 24 bit per pixel. 4) Image collector: This device is a system-integrated structural component that centrally installs standard light sources, cameras, and cuvettes. 5) Host system: The host system uses an Intel quad-core processor, 4 G memory, 32 G solid-state hard disk system, runs the general Windows system, and is equipped with a software system. 6) Touch screen system: An embedded industrial resistive touch screen that measures 7 inches (17.78 cm), with a resolution of 1,024 × 600 and a high-definition industrial screen. It exhibits the advantages of shock resistance, vibration resistance, and high and low temperature resistance.

#### 2.1.2 System software design

The system software is composed of functional modules, such as user information, image acquisition, image processing, and image recognition. The wireless network enables the host computer to connect remotely to the server for data transmission. The system software processes and recognizes the collected images. Users can operate the software more conveniently through human–computer interaction in the touch screen system.

The functions of each module of the system software are as follows. 1) The primary function of the user information module is to record and manage the basic information of users. 2) The primary function of the image acquisition module is to transmit the image files captured by the camera to the software for real-time processing and identification. 3) The image processing module has basic image processing functions, such as region of interest extraction, smoothing denoising, bilateral denoising, and morphological processing. It can select images in key areas. It eliminates noise that may cause interference on an image while retaining the color information of the image to the greatest extent. Consequently, it improves image quality and makes an image easy to identify. 4) The image recognition module can identify the RGB value of image color in the key area and then convert the RGB color space model into the L*a*b* color space model. The hydration level that corresponds to the image is analyzed in accordance with the b* value. Then, the system provides the corresponding rehydration guidance on the basis of the hydration level.

### 2.2 System design principles

#### 2.2.1 Principle of image acquisition

**FIGURE 3:**
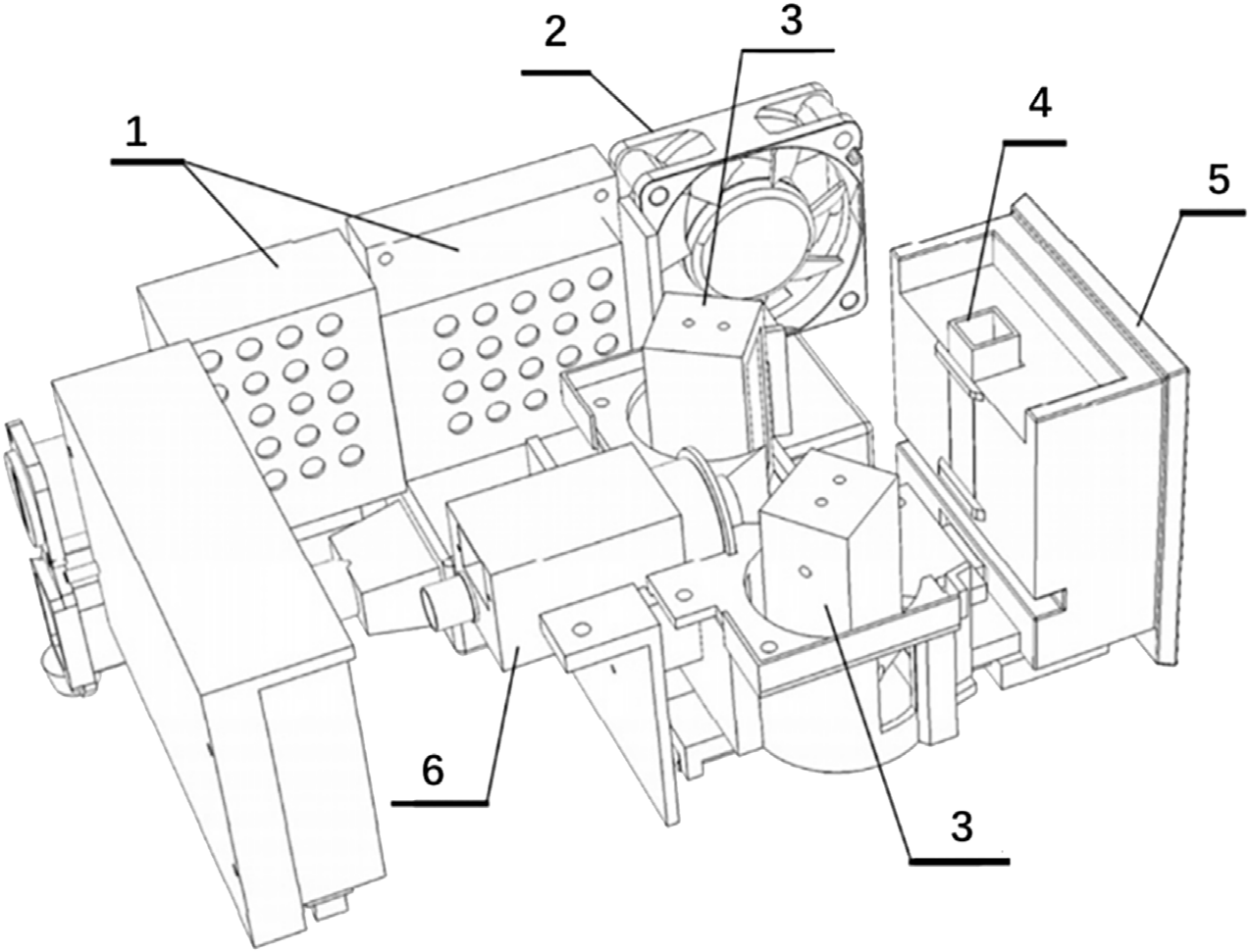
Schematic of image acquisition related structure. No. 1 refers to the industrial power supply. No. 2 refers to the cooling fan. No. 3 refers to the LED surface light source. No. 4 refers to the cuvette. No. 5 refers to the cuvette bracket. No. 6 refers to the camera.

The system completes image acquisition through its components, including the LED standard light source, camera, and cuvette (Figure 3). The camera collects the color information of the urine in the cuvette, and the software extracts and analyzes the color information of the image. Cameras project optical signals onto an image sensor, convert the optical signals into electrical signals, and then into digital image signals. After the assembly of all hardware such as cameras and light sources, we use equipment to test standard samples to ensure that the results of multiple tests on the same sample are consistent. The standard curve is formed inside the software by determination of the standard sample. When the test sample is determined, the software can automatically correct the test value according to the standard curve.

The optical principle of liquid color imaging is based on the transmission of light. When light from a standard light source is transmitted onto urine, urine selectively absorbs light of different wavelengths. Using yellow as an example, urine selectively absorbs light of other colors, and only the yellow light is absorbed the least. Therefore, the yellow light is transmitted to the camera, and the urine appears yellow.

#### 2.2.2 Color space model conversion algorithm

After the camera captures the image, the system software analyzes the RGB value of the image in the key area and then converts the RGB color space model into the L*a*b* color space model. Then, in accordance with the b* value, the hydration level that corresponds to the urine sample is analyzed. The algorithm for converting the RGB color space model into the L*a*b* color space model is as follows (Chung et al., 2014):

(1) First, the *RGB* color space model is converted into the *XYZ* color space model:

$$\left[\begin{array}{c} X\\ Y\\ Z \end{array}\right]=\left[\begin{array}{ccc} 0.4124 & 0.3575 & 0.1804\\ 0.2126 & 0.7151 & 0.0721\\ 0.0193 & 0.1191 & 0.9502 \end{array}\right]\left[\begin{array}{c} R\\ G\\ B \end{array}\right]$$ (1)

(2) Then, the *XYZ* color space model is converted into the *L∗;a∗b∗* color space model:

$$\begin{array}{cc} L* & =116f\left(Y/Y_{n}\right)-16\\ a* & =500\left[f\left(X/X_{n}\right)-f\left(Y/Y_{n}\right)\right]\\ b* & =200\left[f\left(Y/Y_{n}\right)-f\left(Z/Z_{n}\right)\right] \end{array}$$ (2)

Note: *Xn* = 95.047, *Yn* = 100.0, and *Zn* = 108.883.

Among them, the calculation formula of *f*(*t*) is as follows:

$$f\left(t\right)=\left\{ \begin{array}{l} t^{1/3}\;if\;t>{\left(\frac{6}{29}\right)}^{3}\;\\ \frac{1}{3}{\left(\frac{29}{6}\right)}^{2}t+\frac{4}{29}\;elsewhere \end{array}\right.$$ (3)

#### 2.2.3 System hydration grading standard

We refer to the team’s research base (Feng et al., 2022) and relevant high-quality literature (Armstrong et al., 1994; Casa et al., 2000; Racinais et al., 2015; Thomas et al., 2016; McDermott et al., 2017) to formulate the system’s hydration grading standard in accordance with the corresponding relationship between urine color’s b* value and the hydration level. We substituted the threshold values of each grade of Usg (1.000, 1.005, 1.010, 1.015, 1.020, 1.025, 1.030, and 1.035) into the linear regression equation (b = −1,220 + 1,220 Usg, *R* ^2^ = 0.657), and calculated the threshold value of each grade of urine color’s b* value. The system calculates the corresponding rehydration volume on the basis of the user’s weight and hydration level (Table 1). Then, the corresponding rehydration guidance is presented in the system’s result report interface. For example, when a user’s hydration level is 7 and his/her weight is 80 kg, the system will prompt: “You are seriously dehydrated. You are recommended to take 800 ml–1,200 ml of sports drinks. Pay attention for a short period while rehydrating. You are recommended to take 100 ml–200 ml of sports drinks every 10 min–20 min. The amount of fluid supplementation can be adjusted appropriately in accordance with the actual situation.”

**TABLE 1:** System hydration grading standard.

Hydration level	Urine specific gravity	Hydration status	Urine color’s b* value	Rehydration guidance
Level 1	1.000–1.005	Well hydrated	0–6.1	You are drinking enough.
Level 2	1.005–1.010	Well hydrated	6.1–12.2	You are drinking enough.
Level 3	1.010–1.015	Minimal dehydration	12.2–18.3	You can add up to 0.25% body weight in sports drinks.
Level 4	1.015–1.020	Minimal dehydration	18.3–24.4	You can add up to 0.25% body weight in sports drinks.
Level 5	1.020–1.025	Significant dehydration	24.4–30.5	You need to add 0.25%–0.5% body weight in sports drink.
Level 6	1.025–1.030	Significant dehydration	30.5–36.6	You need to add 0.5%–1% body weight in sports drink.
Level 7	1.030–1.035	Serious dehydration	36.6–42.7	You need to add 1%–1.5% body weight in sports drink.
Level 8	> 1.035	Serious dehydration	42.7–127	You need to add 1.5%–2% body weight in sports drink.

### 2.3 System operation process

**FIGURE 4:**
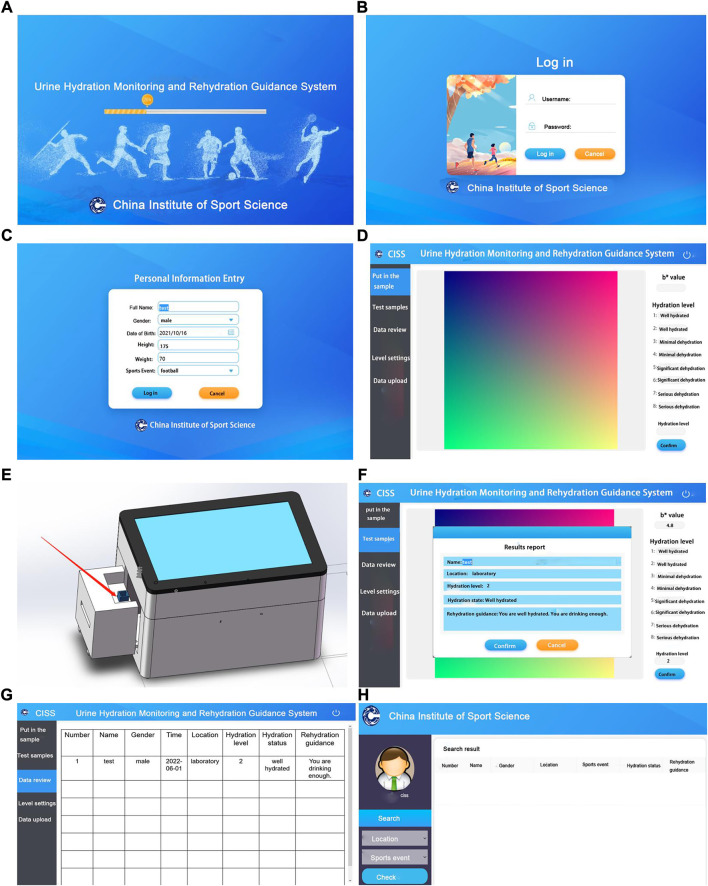
Schematic of the system interface and operation. **(A)** System entry interface, **(B)** login interface, **(C)** personal information entry interface, **(D)** sample insertion interface, **(E)** schematic of placing cuvette, **(F)** test sample and result report interface, **(G)** data upload and review interface, and **(H)** cloud data review interface.

The system operation process includes starting the system, filling in basic information, putting the sample, testing the sample, local data review, local data upload, and cloud data review (Figure 4).

The specific operation process of the system is as follows. 1) Start the system: The system is connected to the power data cable. The user starts the software system and enters the account number and password (Figures 4A,B). 2) Fill in the basic information: The user fills in the required information, such as “full name, height, weight, gender, date of birth, and sports events” (Figure 4C). 3) Put the sample: After the user cleans the cuvette with filter paper, a plastic straw is used to drop urine into the cuvette until about 2/3 of its capacity. The user drags out the bracket and loads the cuvette into the slot. Then, the user pushes the bracket back to its original position, seals the image acquisition system, and clicks “Place sample” (Figures 4D,E). 4) Detecting the sample: After the user puts the sample, the system’s touch screen will display the image of the cuvette. Then, the user clicks “Test sample”, fills in the “Location”, checks the result report, and stores the test information. If the next sample still needs to be tested, then the user must clean the cuvette, reinsert the sample, and then test the sample (Figure 4F). 5) Local data review: After detection, the user can click “Data review” to view the historical test data of the system (Figure 4G). 6) Local data upload: When the system is connected to the network, users can upload local data to the cloud platform system (Figure 4G). 7) Cloud data review: The Internet cloud platform system forms a big data system by receiving detection data from each device. The user logs in to the system’s website, enters his/her account number and password, and then views his/her historical data. Users can filter target data by clicking on different sports events and locations. Users can copy the data for further processing (Figure 4H).

## 3 Results and discussion

### 3.1 System testing

To test the stability and validity of the system, we collected the morning urine of 100 healthy high-level young athletes (age: 24.08 ± 3.73 years, height: 168.32 ± 13.36 cm, body mass: 61.05 ± 19.68 kg, 58 males/42 females), and used the system to determine the hydration level of the urine samples. We used IBM SPSS Statistics version 26.0 for data analysis. Statistical significance was accepted at *p* < 0.05.

#### 3.1.1 System stability test

We repeated the detection of urine samples two times and conducted Pearson correlation analysis. Then, we found that the hydration levels of the two detection processes were highly correlated (r = 1, *p* < 0.01). The results suggested that system stability was high.

#### 3.1.2 System validity test

**FIGURE 5:**
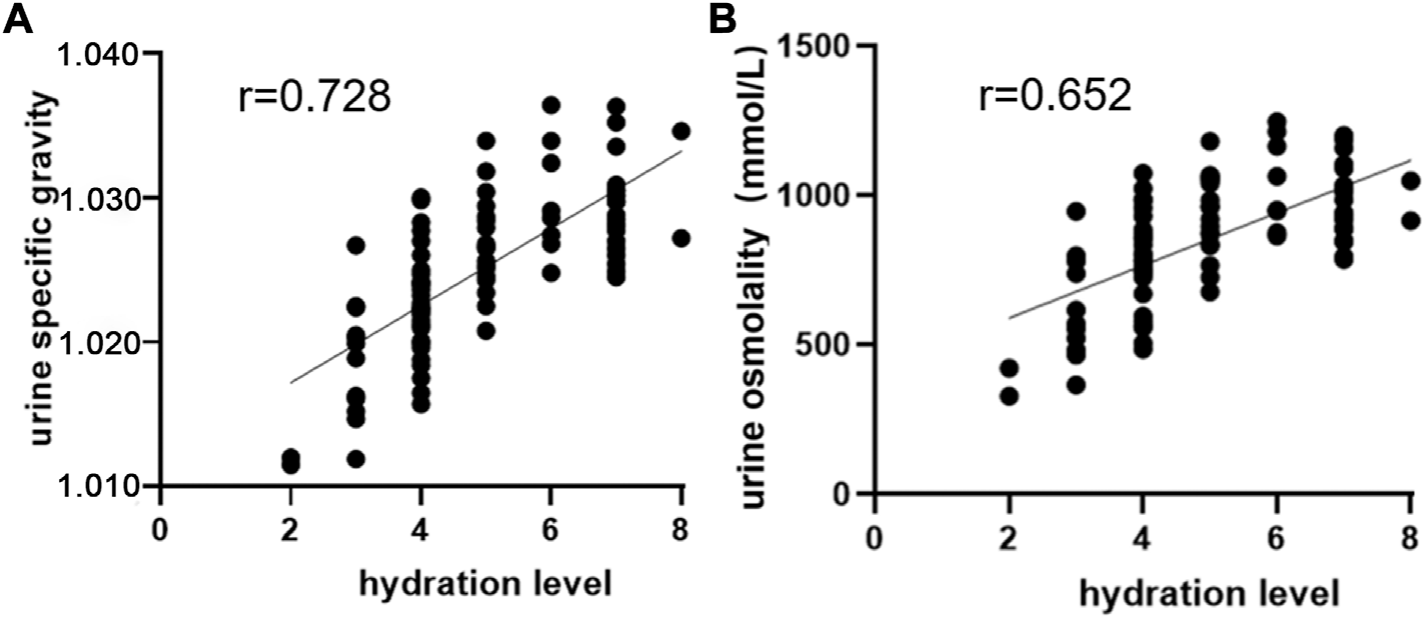
Correlation analysis of hydration level with Usg and urine osmolality. **(A)** Correlation analysis between hydration level and Usg. **(B)** Correlation analysis between hydration level and urine osmolality.

The system is the first device used to monitor hydration level on the basis of urine color’s L*a*b* parameters. Its detection principle is not the same as those of other hydration monitoring devices. At present, no “gold standard” for assessing hydration status has been accepted (Armstrong, 2007). By using Usg and urine osmolality as reference standards, we evaluated the validity of the system by analyzing the correlation between hydration level and Usg and urine osmolality. We used the system to test hydration levels, a digital refractometer (PAL-10S, Atago, Tokyo, Japan) to test Usg, and a vapor osmometer (Vapro 5,600, Wescor, United States) to test urine osmolality. Pearson correlation analysis was adopted for data. The results showed that hydration level exhibited a high correlation with Usg and urine osmolality (r = 0.728, 0.652, *p <* 0.01), indicating that the system had relatively high validity (Figure 5).

### 3.2 Strengths and limitations of the system

#### 3.2.1 Strengths of the system

The strengths of the system are as follows. 1) At present, relevant hydration monitoring equipment adopts the RGB color space model. For the first time, we use the L*a*b* color space model to develop hydration monitoring and rehydration guidance equipment. The L*a*b* color space model covers all the colors that the human eyes can see, and it is currently the color space model with the widest color gamut. 2) This system uses urine color’s b* value as a test index for the first time, introducing a new hydration assessment method. The detection method of this system is noninvasive and safe, and the cost is low. 3) The system strictly controls the test environment for urine color. Compared with the traditional visual urine color chart, the system’s test results are unaffected by the operator’s subjectivity and the external test environment. The system exhibits stable performance, high precision, a simple and friendly interface design, easy operation, and fast data analysis. It can distinguish different hydration states and provide real-time results. 4) The system is equipped with a 10 mm optical path quartz cuvette that is commonly sold in the market, and it presents good light transmittance. The amount of urine required for the system test is small. 5) The system cannot only save data locally but also transmit local data to the cloud for storage, realizing the long-term, permanent, and massive storage of test data. The system is capable of testing large sample sizes. System data can be processed and analyzed by researchers. 6) By using this system, athletes can have an intuitive visual perception of urine color and hydration level. Personalized rehydration guidance can also improve the enthusiasm and awareness of rehydration. 7) The appearance, hardware and software design of this system are unique and innovative. Compared with other urine hydration monitoring systems, this system has the function of rehydration guidance and standardized operation process.

#### 3.2.2 Limitations of the system

The limitations and recommendations of the system are as follows. 1) The design of this system comprehensively considers factors, such as component cost, technical level, research, and development cycle, and funding. At present, the system is slightly large. In the future, the hardware can be designed to be more portable, and the system can be further updated to produce and popularize the system. 2) At present, no unified “gold standard” is available for assessing hydration (Armstrong, 2007). Therefore, we propose to integrate a variety of test indicators and methods for evaluating the hydration of athletes to achieve complementary advantages. 3) This system uses the urine color parameter as the evaluation index of hydration, and urine color may be affected by confounding factors, such as fluid intake, strenuous exercise, food, medicines, and diseases (Zubac et al., 2018). These factors may result in urine color that does not accurately identify the body’s true hydration. Therefore, we recommend that athletes should minimize strenuous exercises and reduce intake of high-pigment fluids and food several hours before urine collection. 4) For the timing of urine collection, spot urine is fresh and readily available, but the test results are less accurate. Therefore, we recommend collecting morning urine sample, which is stable and the test results are easy to standardize (Baron et al., 2015; Cheuvront et al., 2015). In addition, urine hydration indicators are stable for up to 7 days in a refrigerated environment (Adams et al., 2017). If the hydration index cannot be tested immediately after collecting the urine sample, the urine sample should be stored under refrigeration and the test should be completed as soon as possible or within 1 week.

## 4 Conclusion

In this paper, we have successfully developed the urine hydration monitoring and rehydration guidance system for athletes based on urine color’s L*a*b* parameters. The system can evaluate the hydration of athletes objectively and accurately. It provides intuitive and personalized rehydration guidance, offering a new method for solving practical problems in sports training. The system has high practical value and broad application prospects. It cannot only serve athletes in the field of competitive sports but also the general population in the fields of public health and medical care.

At present, the development of hydration monitoring system has been one of the research focuses. Athletes, soldiers, workers, children, pregnant women, the elderly, and ordinary adults all need timely hydration monitoring. For the first time, we took the L*a*b* parameters of urine color as test indicators, and successfully developed a urine hydration monitoring system. The ideal hydration monitoring equipment should be simple, fast, safe, portable, low-cost, non-invasive, cheap, easy to operate, effective, accurate and practical. In the future, we can combine multiple disciplines, adopt more new indicators and technologies, and develop various types of equipment to meet the needs of different populations and scenarios.

## Data Availability

The raw data supporting the conclusions of this article will be made available by the authors, without undue reservation.

## Abbreviations

Usg, urine specific gravity.
